# Body Mass Index and Blood Pressure Screening in a Rural Public School System: the Healthy Kids Project

**Published:** 2006-09-15

**Authors:** William E Moore, Aietah Stephens, Terry Wilson, Wesley Wilson, June E Eichner

**Affiliations:** University of Oklahoma Prevention Research Center; University of Oklahoma Prevention Research Center, Oklahoma City, Okla; University of Oklahoma Prevention Research Center, Oklahoma City, Okla; University of Oklahoma Prevention Research Center, Oklahoma City, Okla; University of Oklahoma Prevention Research Center, Oklahoma City, Okla

## Abstract

**Introduction:**

All students (N = 2053) in Anadarko public schools, grades kindergarten through 12, were invited to be screened for height, weight, and blood pressure to assess the health status of this multiracial, multiethnic (American Indian, white, African American, and Hispanic) population in southwestern Oklahoma.

**Methods:**

The Centers for Disease Control and Prevention's 2000 growth charts were used to determine body mass index (BMI) percentiles, and standards from the National High Blood Pressure Education Program Working Group on Hypertension Control in Children and Adolescents were used to assess blood pressure.

**Results:**

Seven hundred sixty-nine students with active consent participated in the screening. Of these, approximately 28% were overweight. American Indians were at significantly greater risk of being overweight or at risk for overweight than whites (relative risk [RR], 1.4; 95% confidence interval [CI], 1.1–1.7) as were African Americans (RR, 1.5; 95% CI, 1.1–2.0), whereas Hispanics (RR, 1.3; 95% CI, 0.9–2.0) did not have a statistically significant increased risk compared with whites. BMI at or above the 95th percentile was strongly associated with elevated blood pressure (≥90th percentile) (RR, 3.8; 95% CI, 2.6–5.4).

**Conclusion:**

Students who participated in this BMI screening in the Anadarko public school system evidenced high rates of excess weight, with American Indians and African Americans at greatest risk. Elevated BMI was strongly associated with elevated blood pressure.

## Introduction

The Healthy Kids Project is an ongoing height, weight, and blood pressure screening project in the Anadarko, Okla, public school system. The project originated from discussions about health issues at a quarterly meeting of the University of Oklahoma Prevention Research Center's (OUPRC's) Community Advisory Board (CAB). A member of the CAB commented that while examining children in her role as a nurse for the public schools, she noticed many overweight children with *acanthosis nigricans*, defined in *The Merck Manual of Diagnosis and Therapy* as "a velvety hyperpigmentation on the neck, axillae, and groin" that "is probably the skin manifestation of severe and chronic hyperinsulinemia" (1). She also suspected that many children might be hypertensive based on blood pressure readings of children she examined for routine health problems. A collaboration among the CAB, OUPRC, and the Anadarko public schools was established to investigate these observations. A plan was implemented to offer screening to all children in the Anadarko public schools to determine the prevalence of overweight and elevated blood pressure. The group decided that the results of this screening should be presented to the administration of the school system to be used in policy decisions related to children's health in the district and that individual results would be given to parents of students who participated in the screening.

The United States has experienced an epidemic increase in the prevalence of adult obesity in the past decade (2-4). The 1999–2000 National Health and Nutrition Examination Survey estimates that approximately 64.5% of adults are overweight (body mass index [BMI] ≥25 kg/m^2^) and that approximately 30.5% are obese (BMI ≥30 kg/m^2^) (5). Children and adolescents have not been spared; the survey indicates that approximately 15.3% of children and 15.5% of adolescents are overweight (BMI ≥95th percentile for age and sex) (5). Analysis of data from the National Longitudinal Survey of Youth shows that some racial groups and geographic areas have even higher prevalence rates of overweight. Hispanic (21.8%) and African American (21.5%) youths had appreciably higher rates than non-Hispanic white (12.3%) youths, and the southern states had the highest prevalence (17.1%) among geographic regions (6). Recent results of the screening of all public school children in Arkansas (21% overweight) and surveys in Texas (20%–21% overweight) also indicate a higher prevalence of overweight in these states (7-9). Although there are many studies from different geographic areas indicating that American Indian children have a greater prevalence of obesity than white children, national data with an appropriate sample do not exist (4,10). 

Given the increases in weight for height and age, accompanying blood pressure elevations may be expected. A recent investigation of public school students aged 10 to 19 years in Houston, Tex, demonstrated that hypertension is associated with excess weight and that the prevalence of hypertension may be increasing. However, this study did not include children aged 5 to 9 years or American Indians (8).

Recent increases in childhood and adolescent obesity have paralleled large increases in childhood and adolescent type 2 diabetes (11-13). This increase portends possible increases in other chronic conditions associated with childhood and adolescent obesity, such as asthma, sleep apnea, and gallbladder disease (11). The importance of stopping the epidemic of childhood obesity also stems from the association of obesity with adult cardiovascular disease (CVD) and type 2 diabetes. Obesity is a risk factor for both diseases. There is evidence that overweight children become overweight adults, and this association becomes stronger the longer obesity persists during childhood. This association suggests that obesity among children is a long-term risk factor and may indicate earlier onset of adult disease (14-17). Being overweight as an adult is also associated with elevated blood pressure, and there are several reports that hypertension is increasing among American Indian adults in the Southwest (18-23).

Other CVD risk factors also cluster with obesity in children. The Bogalusa Heart Study showed strong positive associations between BMI, blood pressure, and low-density lipoprotein cholesterol (LDL-C) and showed a negative association between BMI and high-density lipoprotein cholesterol (HDL-C) (24). 

The Anadarko School District provides an exceptional opportunity to examine the relationship between weight and blood pressure status among American Indian, white, Hispanic, and African American students living in the same rural community. This investigation can generate data of broad scientific interest while also providing much needed information for the school district, students, and their parents.

## Methods

### Setting

The Anadarko School District, in southwestern Oklahoma, has a total population of 9370. The 2000 U.S. Census, using mutually exclusive racial and ethnic categories, indicates that 41.9% percent of respondents identified themselves as white, 37.9% as American Indian, 5.3% as African American, 0.2% as Asian, 6.3% as multiracial, and 8.4% as Hispanic or Latino. The school-aged population (5–19 years) represented 40.3% of the American Indian population and 19.3% of the white population (25). The median 1999 household income in the school district was $26,540, approximately 79% of the Oklahoma median and 63% of the U.S. median (26). American Indian, African American, and Hispanic households had 83%, 86%, and 74%, respectively, of the income of white households (25).

### The Anadarko public school system

The public school system consists of six schools: three elementary schools (one for kindergarten and first grade, one for second and third grades, and one for fourth and fifth grades), one middle school (sixth through eighth grades), one high school (ninth through twelfth grades), and one alternative high school. Approximately 2053 students were enrolled during the 2001 to 2002 school year. The racial and ethnic composition of the student body was 60.5% American Indian, 28.8% white, 4.8% African American, and 5.8% Hispanic or Latino. Girls comprised 49.4% of the student body and boys 50.6%. Seventy-seven percent of the students were eligible for free or reduced-price lunches in 2001, 1.58 times the Oklahoma average (27).

### Participants

We recruited participants from the Anadarko student population during the annual enrollment day for the 2001 to 2002 school year. A recruitment letter and consent/assent form were included in the enrollment package that each parent or guardian is required to pick up and complete before the start of each school year. Students were also recruited through distribution of the letter and form at each school while screening was being conducted. We returned to each school until all those with consent/assent forms were screened or we were notified that they had moved. Active parental or guardian consent and child assent were obtained in accordance with the project protocol approved by the University of Oklahoma's Health Sciences Center Institutional Review Board. Data were collected on 769 students.

### Measures

Height was measured to the nearest 0.1 centimeter using a SECA Road Rod stadiometer, and weight was measured to the nearest 0.1 kilogram using a SECA 770 digital scale (SECA Corp, Hanover, Md). Both height and weight were measured with shoes, coats, and other heavy outerwear removed. BMI was calculated as weight in kilograms divided by height in meters squared. One investigator measured the height and weight of approximately 85% of the participants, and all field measurements were obtained by individuals trained and supervised by this investigator. BMI and sex- and age-specific BMI percentiles were calculated using the Centers for Disease Control and Prevention's (CDC's) Epi Info software and its 2000 growth chart database (28). The BMI percentiles were stratified into sex- and age-specific classifications based on CDC's 2000 growth charts as follows: underweight (<5th percentile), normal weight (≥5th percentile but <85th percentile), at risk of overweight (≥85th percentile but <95th percentile), and overweight (≥95th percentile).

Blood pressure was categorized according to the classifications described by the 1996 National High Blood Pressure Education Program Working Group on Hypertension Control in Children and Adolescents Report (29). The classifications are defined according to the following age, sex, and height specific blood pressure percentiles from normative tables based on one blood pressure measurement: normal (systolic and diastolic blood pressure <90th percentile), high normal (systolic or diastolic ≥90th percentile but <95th percentile), and hypertensive (systolic or diastolic ≥95th percentile). Classification is based on the highest categorization of either systolic or diastolic blood pressure. The report also states that a diagnosis of hypertension should be based on the average of at least two measurements per measurement occasion on at least three separate occasions over weeks or months and that a mercury sphygmomanometer should be used. It is inferred that an assessment of normal blood pressure requires only one measurement.

Blood pressure was measured using an Omron HEM-907 IntelliSense (Omron Healthcare, Inc, Vernon Hills, Ill) electronic digital blood pressure monitor with appropriately sized cuff. Blood pressure measurement began after the student sat quietly for 3 minutes. This measurement was recorded. For participants younger than 18 years, age-, sex-, and height-specific tables for the 90th (high normal) and 95th (hypertensive) percentile systolic and diastolic blood pressures were consulted to determine blood pressure classification (29). Students were considered to have elevated blood pressure if their systolic or diastolic blood pressure was in at least the 90th percentile. If the student's systolic and diastolic blood pressure were normal, blood pressure was recorded, and the student's blood pressure measurement was concluded. However, the blood pressure was retaken after 1 minute if the first measurement was elevated. The process was repeated one more time if the blood pressure remained elevated. Some elevated blood pressures were missed during the process of examining blood pressure tables in a field setting; however, these individuals were retested in a subsequent session. If the blood pressure was elevated for consecutive measurements, the student's blood pressure was remeasured at least 2 weeks later. If this measurement also revealed consecutive elevated blood pressure, the student was scheduled for a third and final blood pressure assessment. The names of students whose blood pressure remained elevated on three separate measurement occasions were given to school nurses for follow-up. Blood pressure for these students was measured in the privacy of the nurse's office using a mercury sphygmomanometer.

This blood pressure screening protocol was designed to increase test specificity by reducing false positives while maintaining sensitivity for referral. It was also designed to allow a practical and sustainable workload that could be continued annually with limited resources. The protocol was designed to investigate the relationship between weight status and blood pressure, not to determine the prevalence of hypertension. Racial and ethnic classification and date of birth were based on self-report and verified with school records for all elementary school students.

### Statistical analyses

Stata version 8.2 (StataCorp LP, College Station, Tex) was used to perform descriptive and inferential statistical analyses. Binomial regression models were generated to explore the associations between weight status and racial and ethnic classification and the relationship between weight status and blood pressure. To ensure that minimal residual confounding existed, we adjusted these models for age and sex. Ninety-five percent confidence intervals (CIs) were generated for all risk ratio estimates. Because of the difficulty in reconciling the child and adolescent blood pressure classification standards with those for adults (18 years and older), blood pressure analyses were performed only on students younger than 18 years (n = 745). Blood pressure classification for each measurement occasion was based on the last blood pressure measurement taken.

## Results

### Response

We screened 769 (37.5%) of 2053 students in the school system for height and weight. All but two students were also screened for blood pressure. The elementary school response rate was 50.8% (517/1017), the middle school response rate was 28.3% (140/494), and the high school response rate was 20.7% (112/542). There was a significant linear trend of decreasing participation with increasing grade level (Cochran-Armitage trend test, *P* < .001). Of the participants, 62.4% were American Indian, 27.3% were white, 6.0% were African American, and 4.3% were Hispanic. One Asian student also participated but was excluded from further analyses. The overall response rate by racial and ethnic classification did not differ significantly from the expected distribution (Χ^2^ goodness-of-fit test, Χ^2^_3_ = 6.3, *P* = .10). This lack of racial or ethnic bias held when the data were stratified by school status (elementary, Χ^2^ goodness-of-fit test, Χ^2^_3_= 1.8, *P* = .61; middle school, Χ^2^_3 _= 5.6, *P* = .13; high school, Χ^2^_3 _= 2.8, *P* = .42). Neither was an overall sex bias in response rates noted (Χ^2^ goodness-of-fit test, Χ^2^_1_ = .09, *P* = .92). This lack of sex bias also held when the data were stratified by school status (elementary, Χ^2^ goodness-of-fit test, Χ^2^_1_ = 0.0, *P* = .86; middle school, Χ^2^_1 _= 0.6, *P* = .43; high school, Χ^2^_1_ = 0.4, *P* = .54).

### Overweight status

Table 1 provides BMI categories by grade level and sex; Table 2 provides BMI categories by race and ethnicity. Binomial regression models to examine the relationship between weight status and race were adjusted for age and sex (Table 3). Overall, 27.9% of the students screened were overweight. American Indians had a higher prevalence of overweight (32.2%) than any other racial or ethnic group. This prevalence was significantly greater than the prevalence for white students when students who were overweight were compared with those of normal or underweight status in a binomial regression model (Table 3). No statistically significant racial or ethnic differences were found for risk of overweight status, but the point estimates for American Indian, African American, and Hispanic students were greater than for white students. A binomial regression model comparing those at risk for overweight or overweight with those of normal or underweight status provides evidence that American Indian and African American students are at significantly greater risk of being more than normal weight than white students (Table 3). Although Hispanic students tended to be heavier than white students, this result was not statistically significant. This comparison is important because risk of becoming overweight and being overweight track with similar strength into adult obesity (15,17). No statistically significant sex differences in weight status were evident; 28.0% of males and 27.8% of females were classified as overweight (Pearson Χ^2^_3_= 2.98, *P* = .40). We found a hint of an increasing linear trend for overweight as grade level increases (Table 1). However, this trend is mitigated by the decrease in overweight seen among high school students when compared with middle school students (Cochran-Armitage trend test, *P* = .11). When the high school results are excluded, there is a statistically significant increasing trend for overweight as grade level increases (Cochran-Armitage trend test, *P* = .003).

### Blood pressure categories

From the total sample of 747 students aged less than 18 years, 745 had their blood pressure measured. Two students were not measured for blood pressure because their clothing interfered with the measurement. No student with elevated blood pressure was lost to follow-up. Only data from the 745 students younger than 18 years were examined further because of the difficulty reconciling adult blood pressure standards with those for children and adolescents. At the first screening session, 18.4% (137/745) of the students had blood pressure at or above the 90th percentile for their age, sex, and height. After the second measurement occasion, this percentage was reduced to 5.1% (38/745), and only 2.8% (21/745) had blood pressure at or above the 90th percentile that persisted for three measurement occasions (i.e., three to nine consecutive measurements.)

### Association of overweight status and elevated blood pressure

A strong association was seen between students with a BMI at or above the 85th percentile and blood pressure at or above the 90th percentile on the first measurement occasion (Table 4). This association gained strength as elevated blood pressure persisted with repeated blood pressure measurements, with the second measurement occasion providing a crude relative risk (RR) of 5.95 (95% CI, 2.52–14.07) and the third a crude RR of 6.70 (95% CI, 1.99–22.55) (data not shown). Small numbers of students with elevated blood pressure prevented regression modeling for these subsequent measurements.

## Discussion

### Response rates

The response rates were reasonable but far from ideal. Volunteer bias may have substantially skewed the observed prevalence rates for overweight. It is possible that this was the case for older students in middle school and high school. A result of this bias might be an underestimation of the prevalence rates for underweight, risk of overweight, and overweight. Weight consciousness and autonomy from parental influence arguably increase with age, and therefore older overweight students may have been less likely to participate in the screening than normal weight students. The cross-sectional decline in overweight prevalence between the middle and high school cohorts also indicates that there was an underrepresentation of overweight students among those screened and that this underrepresentation may have increased with age. The apparent decline could also be a cohort effect; however, anecdotal evidence from school personnel indicates that many overweight students in high school and middle school did not participate in the screening. Recent statewide results from Arkansas may indicate that a reduction in overweight among high school students is a real phenomenon, but the decline is not discussed (7).

Low to moderate response rates could lead to biased prevalence rates for elevated blood pressure.  An underrepresentation of overweight students could definitely have this effect, but despite low response rates, the association between elevated blood pressure and elevated weight should remain unbiased.

### Overweight status

The results of the weight status screening provide important information for the participants, parents, and school district. The prevalence of overweight among the participants is nearly twice that of recent national rates, rates already considered epidemic (5). The prevalence of overweight among the American Indian participants is more than twice the national prevalence estimates for the general school-aged population. Even among white participants, usually seen as at less risk for overweight than American Indians, African Americans, or Hispanics, the prevalence of overweight was greater than national rates for children and adolescents. The overweight prevalence rate for white participants (18.1%) was the lowest by racial and ethnic classification but was still substantially greater than recent national prevalence estimates of approximately 15.4% for children and adolescents and much greater than the *Healthy People 2010* objective of less than or equal to 5% (5,30).

A unique aspect of the overweight prevalence data for the Anadarko public schools is that it provides an opportunity to compare the overweight status of American Indian and white students living in the same integrated community. Although many potential confounders exist, it is notable that American Indian students had a significantly increased risk of being overweight compared with their white peers (Table 3). Explanations for this difference are yet to be determined. Biological and cultural differences are strong possibilities (4). Economic factors may also play a role; whereas 54% of white students were eligible for free or reduced-price lunches, 85% of American Indian, 85% of African American, and 90% of Hispanic students were eligible. However, a recent large-scale investigation has shown that food insecurity, a corollary of poverty, is associated with lower BMIs in children (31). Another recent investigation has provided evidence that community availability of fitness facilities and other venues for activity is positively associated with healthy weight (32). However, the fact that the students all live in the same community diminishes the possibility that access to sidewalks, parks, playgrounds, fast-food outlets, or grocery stores is an explanatory factor.

### Blood pressure status

Only 2.8% of the students had elevated blood pressure (≥90th percentile) that persisted over three measurement sessions and multiple blood pressure readings. However, the National High Blood Pressure Education Program Working Group on Hypertension Control in Children and Adolescents stated that it should be expected that less than 1% of children and adolescents will be diagnosed as hypertensive when multiple measurements are taken over weeks or months. Doubling this expected percentage to extrapolate to the 90th percentile indicates that there may be an excess prevalence of elevated blood pressure among these students. However, the unique protocol and electronic blood pressure monitors used for this screening may underestimate the prevalence of elevated blood pressure compared to using averaged measurements with a mercury sphygmomanometer.

Because of the low yield from screening children for hypertension, recommendations against universal screening have been made (33,34). However, these recommendations were based on the need for highly trained personnel using mercury sphygmomanometers. The advent of high-quality electronic blood pressure monitors, combined with the epidemic of obesity, may have changed the cost–benefit equation for blood pressure screening. In fact, recent research supports the premise that rates of hypertension may be increasing among children but that normative data based on electronic oscillometric blood pressure measurement are needed for future screening efforts (8).

### Association of overweight status and elevated blood pressure

Although the prevalence of elevated blood pressure in the Anadarko public schools is difficult to interpret, the consistent and strong association of high BMI with elevated blood pressure may presage future increases in child, adolescent, and adult hypertension if the prevalence of obesity is sustained or increases (20,35).

### Impact

We presented the data in this paper to the principals, superintendent, and other interested school personnel of the Anadarko public school system. Their response has been positive and demonstrative of the often-forgotten altruism inherent in America's public schools. The middle school implemented a school-based walking program for sixth graders in the 2002 to 2003 school year. The high school implemented and evaluated a school-based student walking program during the second half of the 2002 to 2003 school year. Lastly, the district has decided to commit resources to perform weight and blood pressure screening annually as part of usual student health activities and to examine cost vs benefits and effectiveness of this activity.

## Figures and Tables

**Table 1:** Weight Status^a^ of Healthy Kids Project Participants (n = 768),^b^ by Grade Level and Sex, Anadarko, Okla, School District, 2001–2002

**Grade**	**Sex**	**Underweight, No. (%)**	**Normal Weight, No. (%)**	**At Risk of Overweight, No. (%)**	**Overweight, No. (%)**	**Total, No. (%)**
K-1	Female	1 (1.2)	42 (50.6)	18 (21.7)	22 (26.5)	83 (45.6)
	Male	0 (0)	59 (59.6)	22 (22.2)	18 (18.2)	99 (54.4)
2-3	Female	1 (1.1)	54 (59.3)	14 (15.4)	22 (24.2)	91 (49.2)
	Male	0 (0)	50 (53.2)	19 (20.2)	25 (26.6)	94 (50.8)
4-5	Female	0 (0)	39 (50.6)	13 (16.9)	25 (32.5)	77 (51.7)
	Male	0 (0)	34 (47.2)	12 (16.7)	26 (36.1)	72 (48.3)
6-8	Female	0 (0)	38 (50.7)	11 (14.7)	26 (34.7)	75 (53.6)
	Male	0 (0)	30 (46.2)	12 (18.5)	23 (35.4)	65 (46.4)
9-12	Female	1 (1.9)	32 (61.5)	9 (17.3)	10 (19.2)	52 (46.4)
	Male	1 (1.7)	25 (41.7)	17 (28.3)	17 (28.3)	60 (53.6)
All grades	Female	3 (0.8)	205 (54.2)	65 (17.2)	105 (27.8)	378 (49.2)
	Male	1 (0.3)	198 (50.8)	82 (21.0)	109 (27.9)	390 (50.8)
Total		4 (0.5)	403 (52.5)	147 (19.1)	214 (27.9)	768 (100)

K indicates kindergarten

a Weight status was categorized as follows: underweight (body mass index [BMI] <5th percentile for age and sex), normal weight (BMI ≥5th percentile but <85th percentile for age and sex), at risk of overweight (BMI ≥85th percentile but <95th percentile for age and sex), and overweight (BMI ≥95th percentile for age and sex).

b Data for one Asian student was excluded from the analysis.

**Table 2:** Weight Status^a^ of Healthy Kids Project Participants (n = 768),^b^ by Race and Ethnicity, Anadarko, Okla, School District, 2001–2002

**Race and Ethnicity**	**Underweight, No. (%)**	**Normal Weight, No. (%)**	**At Risk of Overweight, No. (%)**	**Overweight, No. (%)**	**Total, No. (%)**
American Indian	2 (0.4)	234 (48.8)	89 (18.6)	154 (32.2)	479 (62.4)
White	2 (1.0)	131 (62.4)	39 (18.6)	38 (18.1)	210 (27.3)
African American	0 (0)	21 (45.6)	11 (23.9)	14 (30.4)	46 (6.0)
Hispanic	0 (0)	17 (51.5)	8 (24.2)	8 (24.2)	33 (4.3)
All	4 (0.5)	403 (52.5)	147 (19.1)	214 (27.9)	768 (100)

a Weight status was categorized as follows: underweight (body mass index [BMI] <5th percentile for age and sex), normal weight (BMI ≥5th percentile but <85th percentile for age and sex), at risk of overweight (BMI ≥85th percentile but <95th percentile for age and sex), and overweight (BMI ≥95th percentile for age and sex).

b Data for one Asian student was excluded from the analysis.

**Table 3:** Binomial Regression Models Comparing Three Categories of Body Mass Index, Healthy Kids Project, Anadarko, Okla, School District, 2001–2002

**Independent Variables**	**Overweight Compared With Normal or Underweight (n = 621)**	**At Risk of Overweight Compared With Normal or Underweight (n = 554)**	**Overweight or At Risk of Overweight Compared With Normal or Underweight (n = 768)**

	**Relative Risk (95% CI)**	**Relative Risk (95% CI)**	**Relative Risk (95% CI)**
Age, y	1.03 (1.00–1.06)	1.01 (0.97–1.05)	1.01 (0.99–1.04)
**Sex**			
Male	1.03 (0.83–1.28)	1.20 (0.91–1.59)	1.07 (0.92–1.24)
Female	Ref	Ref	Ref
**Race and ethnicity**			
American Indian	1.80 (1.33–2.45)	1.19 (0.86–1.66)	1.39 (1.14–1.70)
African American	1.81 (1.11–2.95)	1.49 (0.85–2.61)	1.48 (1.08–2.04)
Hispanic	1.48 (0.79–2.79)	1.36 (0.72–2.58)	1.33 (0.90–1.97)
White	Ref	Ref	Ref

CI indicates confidence interval; Ref, reference group.

**Table 4:** Binomial Regression Model for Blood Pressure at or Above 90th Percentile: First Measurement Occasion, Healthy Kids Project, Anadarko, Okla, School District (n = 745), 2001–2002

**Independent Variables**	**Relative Risk (95% CI)**
Age, y	1.01 (0.97–1.06)
Sex	
Male	1.25 (0.93–1.67)
Female	Ref
Race and ethnicity	
American Indian	0.75 (0.54–1.03)
African American	0.48 (0.21–1.08)
Hispanic	1.05 (0.57–1.93)
White	Ref
Weight status	
Overweight	3.76 (2.64–5.37)
At risk of overweight	1.87 (1.18–2.96)
Normal or underweight	Ref

CI indicates confidence interval; Ref, reference group.
